# Deficiency of TYROBP, an adapter protein for TREM2 and CR3 receptors, is neuroprotective in a mouse model of early Alzheimer’s pathology

**DOI:** 10.1007/s00401-017-1737-3

**Published:** 2017-06-13

**Authors:** Jean-Vianney Haure-Mirande, Mickael Audrain, Tomas Fanutza, Soong Ho Kim, William L. Klein, Charles Glabe, Ben Readhead, Joel T. Dudley, Robert D. Blitzer, Minghui Wang, Bin Zhang, Eric E. Schadt, Sam Gandy, Michelle E. Ehrlich

**Affiliations:** 1 Department of Neurology, Icahn School of Medicine at Mount Sinai, New York, NY 10029 USA; 2 Department of Biochemistry, Northwestern University, Chicago, IL 60611 USA; 3 Department of Biochemistry and Molecular Biology, University of California at Irvine, Irvine, CA 92697 USA; 4 Department of Genetics and Genomic Sciences, Icahn Institute of Genomic Sciences, Icahn School of Medicine at Mount Sinai, New York, NY 10029 USA; 5 Department of Neuroscience, Icahn School of Medicine at Mount Sinai, New York, NY 10029 USA; 6 Department of Psychiatry and Alzheimer’s Disease Research Center, Icahn School of Medicine at Mount Sinai, New York, NY 10029 USA; 7 Department of Pediatrics, Icahn School of Medicine at Mount Sinai, New York, NY 10029 USA

**Keywords:** TYROBP/DAP12, TREM2 adapter, CR3 adapter, Alzheimer’s disease, Microglia, APP/PSEN1

## Abstract

Conventional genetic approaches and computational strategies have converged on immune-inflammatory pathways as key events in the pathogenesis of late onset sporadic Alzheimer’s disease (LOAD). Mutations and/or differential expression of microglial specific receptors such as TREM2, CD33, and CR3 have been associated with strong increased risk for developing Alzheimer’s disease (AD). DAP12 (DNAX-activating protein 12)/TYROBP, a molecule localized to microglia, is a direct partner/adapter for TREM2, CD33, and CR3. We and others have previously shown that *TYROBP* expression is increased in AD patients and in mouse models. Moreover, missense mutations in the coding region of *TYROBP* have recently been identified in some AD patients. These lines of evidence, along with computational analysis of LOAD brain gene expression, point to DAP12/TYROBP as a potential hub or driver protein in the pathogenesis of AD. Using a comprehensive panel of biochemical, physiological, behavioral, and transcriptomic assays, we evaluated in a mouse model the role of TYROBP in early stage AD. We crossed an Alzheimer’s model mutant *APP* ^*KM670/671NL*^ */PSEN1* ^*Δexon9*^ *(APP/PSEN1)* mouse model with *Tyrobp* ^−*/*−^ mice to generate AD model mice deficient or null for TYROBP (*APP/PSEN1; Tyrobp* ^+*/*−^ or *APP/PSEN1; Tyrobp* ^−*/*−^). While we observed relatively minor effects of TYROBP deficiency on steady-state levels of amyloid-β peptides, there was an effect of *Tyrobp* deficiency on the morphology of amyloid deposits resembling that reported by others for *Trem2* ^−*/*−^ mice. We identified modulatory effects of TYROBP deficiency on the level of phosphorylation of TAU that was accompanied by a reduction in the severity of neuritic dystrophy. TYROBP deficiency also altered the expression of several AD related genes, including *Cd33*. Electrophysiological abnormalities and learning behavior deficits associated with *APP/PSEN1* transgenes were greatly attenuated on a *Tyrobp*-null background. Some modulatory effects of TYROBP on Alzheimer’s-related genes were only apparent on a background of mice with cerebral amyloidosis due to overexpression of mutant *APP/PSEN1*. These results suggest that reduction of *TYROBP* gene expression and/or protein levels could represent an immune-inflammatory therapeutic opportunity for modulating early stage LOAD, potentially leading to slowing or arresting the progression to full-blown clinical and pathological LOAD.

**Electronic supplementary material:**

The online version of this article (doi:10.1007/s00401-017-1737-3) contains supplementary material, which is available to authorized users.

## Introduction

Conventional wisdom has held that the chronic neuroinflammation associated with LOAD may be a secondary or even protective event that occurs in response to Aβ deposition and may occur only in late stages of AD. However, recent genetic and genomic approaches, as well as computational strategies, have converged on immune-inflammatory pathways as risk factors and as key events in the pathogenesis of late-onset sporadic Alzheimer’s disease (LOAD) [19]. Moreover, correlation between inflammatory genes and clinical presentation of previously asymptomatic cerebral amyloidosis (ACA) indicates a role for inflammation and microglia in the progression from ACA to the earliest stages of mild cognitive impairment (MCI) and/or mild clinical AD. Among the genes implicated by the largest available genome-wide association studies [43], one-third is either unique to, or enriched in, microglia. Recently identified mutations and variants in genes encoding important immune receptors including *CD33*, *CR3* (Complement Receptor 3), and *TREM2* (Triggering Receptor Expressed On Myeloid Cells 2), have been genetically linked to LOAD risk, highlighting the potential role of a dysregulated immune response in an early, and perhaps causative role in AD pathogenesis. Unlike autosomal dominant familial Alzheimer’s mutations that promote elevation of the Aβ42:40 ratio or of other variant hyperaggregatable Aβ species, these AD risk factors specify some of the cell surface signal transduction pathways that modulate the phagocytic machinery of microglia.

TYROBP (TYROsine kinase Binding Protein) (also known as DAP12), is a microglial transmembrane signaling polypeptide that contains an immunoreceptor phosphotyrosine-based activation motif (ITAM) in its cytoplasmic domain and is a direct partner/adaptor for immune receptors, including TREM2, CR3, and SIRPβ1 (Signal Regulatory Protein β1) all of which are independently linked to, or associated with, LOAD [5, 7, 23, 55, 86]. Interaction of TYROBP with its partners forms phagocytosis “active zones” (known as phagocytic synapses) on the surface of microglia. In preparation for phagocytosis, there is a respiratory burst that generates reactive oxygen species (ROS) and appears to involve an interaction between TYROBP and CR3, which in turn interacts with complement component C3 associated with nearby neurites. Mice lacking the complement receptor CR3 or expressing defective TYROBP show reduced ROS production and apoptosis [77]. A recent report demonstrates that the complement pathway can mediate the toxic effects of soluble Aβ on synapses, and that overactivation of this pathway in AD leads to excessive synapse pruning and early synapse loss [25]. Since the discovery of a link between mutations of *TREM2* and AD, several studies have emerged regarding the role of a loss of function of TREM2 in AD. While these studies have some conflicting results, the most consistent observation is that either *Trem2* deficiency or *Tyrobp* deficiency can cause reduced recruitment of microglial cells around Aβ plaques. The impact of this reduction in microglia per plaque was interpreted as deleterious in *Trem2* haploinsufficient and *Trem2* deficient mice.

Through a multi-scale integrated computational approach, we and two other independent groups [12, 48, 86] have previously reported *TYROBP* as a network hub or driver gene in LOAD. Additionally, missense mutations in *TYROBP* have been recently reported as risk factors for AD [61]. Evidence associating *TYROBP* to LOAD notwithstanding, it is important to recognize that most *TYROBP* mutations (as well as *TREM2* mutations) represent loss-of-function mutations that result not in AD but in an osteopathy/encephalopathy known as Nasu–Hakola disease (NHD) [59]. One formulation of these data is that the pathogenic mechanism(s) of loss-of-function (nonsense) mutations in *TYROBP* associated with NHD may cause molecular events that differ from those associated with missense polymorphisms that increase the risk for AD.

Herein, we report the effects of a constitutively null mutation in *Tyrobp* on the phenotype of an *APP/PSEN1* mouse model of AD. In the *Tyrobp*-null mouse, there is a deletion of exons 3 and 4 resulting in loss of function of the TYROBP protein by deletion of the transmembrane region and part of the cytoplasmic region including the first tyrosine of the ITAM motif [2]. The *APP/PSEN1* mouse model [29] expresses *APP* ^*KM670/671NL*^ */PSEN1* ^*Δexon9*^ in neurons and accumulates in the interstitial spaces of the brain fibrillar amyloid that goes on to form typical amyloid plaques accompanied by neuritic dystrophy, age-dependent synaptic loss without neuronal loss, and abnormalities in spatial memory [25, 29, 40, 41]. Since *TYROBP* expression is increased in the LOAD brain [86], we hypothesized that the *APP/PSEN1* phenotype may be improved in the presence of reduced TYROBP levels. Since *Tyrobp* is not expressed in neurons, our observations in this report describe non-cell autonomous effects wherein signals arising from microglia perturb the homeostasis of nearby neurons or nerve terminals or the pathophysiology of evolving structural intraneuronal or extracellular Alzheimer’s pathology.

## Methods

### Mouse husbandry

The experimental procedures were conducted in accordance with NIH guidelines for animal research and were approved by the Institutional Animal Care and Use Committee (IACUC) at Icahn School of Medicine at Mount Sinai. *APP* ^*KM670/671NL*^ */PSEN1* ^*Δexon9*^ (*APP/PSEN1)* and *Tyrobp* knockout (KO) mice were obtained from Jackson Laboratories and Taconic/Merck Laboratory, respectively. *APP/PSEN1* mice were crossed with *Tyrobp* KO mice to obtain *APP/PSEN1* mice heterozygous or KO for *Tyrobp*. Four-month-old male and female mice were killed by decapitation. One hemisphere was collected for immunohistochemical analysis. The second hemisphere was collected for transcriptomic and biochemical analyses.

### Immunohistochemical and biochemical analyses

Immunohistochemical and biochemical characterization were performed as previously described [40, 41, 44, 76]. For biochemical analysis, hemibrains were processed via differential detergent solubilization to produce TBS-soluble, Triton-X-soluble, and formic-acid soluble Aβ fractions. For analysis of native oligomeric Aβ peptides, 2 μl protein samples from the TBS-soluble fraction were spotted onto activated/pre-wetted PVDF membrane (0.22 μm; Millipore, Billerica, MA). Membranes were incubated with rabbit pAb A11 (anti-prefibrillar oligomers, 0.5 μg/ml), rabbit pAb OC (anti-fibrillar oligomers and fibrils; 0.25 μg/ml), and mouse mAb Nu-4 (anti-oligomers; 1 μg/ml) [44, 76]. Normalization to total APP/Aβ signal was achieved by detection of human APP transgene metabolites with the mouse pAb 6E10 antibody (1:1000; Covance, Princeton, NJ). To quantify total Aβ levels, human/rat Aβ 1–40/1–42 ELISA kits (Wako) were used according to the manufacturer’s instructions.

For immunohistochemistry, 30 µm thick sagittal sections were incubated with the following antibodies: rabbit anti-Iba1 (1:500; Wako, Richmond, VA), mouse anti-6E10 (1:1000; Covance, Princeton, NJ), and rat anti-CD68 (1:200, mca1957, AbD Serotec BioRad). Sections were then incubated with the appropriate secondary antibody: anti-rabbit Alexa Fluor 488 or Alexa Fluor 568 (1:400; Invitrogen, Carlsbad, CA), anti-mouse Alexa Fluor 568 (1:400; Invitrogen, Carlsbad, CA), and anti-rat Alexa Fluor 488 (1:400; Invitrogen, Carlsbad, CA) antibodies. ThioflavinS (Sigma-Aldrich, T1892, 1% w/v stock solution) was used for labeling amyloid deposits.

For measuring microglia number, Iba1-immunolabeled sections were thresholded and particles analyzed with Fiji (v2.0.0). Sizes of 6E10 immunoreactive plaques and fluorescent intensities were analyzed with Fiji (v2.0.0). The regions of interest were determined by manual tracing. Thioflavin S fluorescence intensity and circularity were analyzed as described [85].

For immunoblotting, membranes were incubated with either anti-CD68 (1:1000, mca1957, AbD Serotec BioRad), anti-phospho-Tau pSer202/Thr205 (1:1000; MN1020, Thermo Fisher Scientific, Waltham, MA), anti-Tau (1:1000; MN1000, Thermo Fisher Scientific, Waltham, MA), anti-Synaptophysin (1:200; ab16659, Abcam, Cambridge, MA), anti-Lamp1 (1:200; ab24170, Abcam, Cambridge, MA), anti-C3 (1:50; ab11862, Abcam, Cambridge, MA), and anti-GAPDH (1:5000; sc32233, Santa Cruz, Dallas, TX) antibodies. Integrated density of immunoreactive bands was measured using MultiGauge Software (FujiFilm). At least two independent western blot analyses were performed and normalized using *APP/PSEN1* female mice as controls.

### Behavior analysis

The Barnes Maze test was performed using a standard apparatus [3, 74]. Four-month-old mice were transported from their cage to the center of the platform via a closed starting chamber where they remained for 10 s prior to exploring the maze for 3 min. Mice failing to enter the escape box within 3 min were guided to the escape box by the experimenter, and the latency was recorded as 180 s. Mice were allowed to remain in the escape box for 1 min before the next trial. Two trials per day during 4 consecutive days were performed. The platform and the escape box were wiped with 70% ethanol after each trial to eliminate the use of olfactory cues to locate the target hole. All trials were recorded by video camera and analyzed with ANY-maze video tracking software (Stoelting Co, Wood Dale, USA).

### Field electrophysiology

Coronal brain slices containing the hippocampal formation were prepared as previously described [17]. Following anesthesia with isoflurane, brains were rapidly removed and cut into 400 µm thick coronal sections using a vibratome VT1000S (Leica Microsystems, Germany). Brain slices were incubated at room temperature for ≥3 h in a physiologic ACSF containing 120 mM NaCl, 3.3 mM KCl, 1.2 mM Na_2_HPO_4_, 26 mM NaHCO_3_, 1.3 mM MgSO_4_, 1.8 mM CaCl_2_, 11 mM Glucose (pH 7.4) and then transferred to a recording chamber perfused with ACSF at a flow rate of ~2 mL/min; experiments were performed at 28.0 ± 0.1 °C. Recordings were acquired with a GeneClamp 500B amplifier (Axon Instruments, Union City, CA) and Digidata 1440A (Molecular Devices, Sunnyvale, CA). All signals were low-pass filtered at 2 kHz and digitized at 10 kHz. For extracellular field recordings, a patch-type pipette was filled with ACSF and placed in the middle third of stratum radiatum in area CA1. Field excitatory postsynaptic potentials (fEPSPs) were evoked by activating Shaffer Collaterals with a Concentric Bipolar Electrode stimulator (FHC, St Bowdoin, ME) placed in the middle third of stratum radiatum 150–200 µm away from the recording pipette. Square-wave current pulses (60 ms pulse width) were delivered through a stimulus isolator (Isoflex, AMPI). Input–output curves were generated by a series of stimuli in 0.1 mA steps. Paired-pulse facilitation was measured by delivering two stimuli at 20, 50, and 100 ms inter-stimulus intervals. Each inter-stimulus interval was repeated three times and the resulting potentials were averaged. The paired-pulse ratio was calculated by dividing the slope of the second EPSP by the slope of the first EPSP. All results were analyzed by ANOVAs followed by Tukey post hoc tests. Baseline recordings (stable for 20 min) were made every 30 s using stimuli that yielded a response equal to 50% of spike threshold. LTD was induced using a 1-Hz train of 900 bursts, each burst containing three stimuli delivered at 20 Hz, using stimulus strength just superthreshold for evoking a population spike during baseline.

### Molecular biological analyses

RNA isolation, library preparation, differential expression analysis and gene set enrichment analyses were performed as described [6, 26, 27, 64, 66, 67].

### Computational screen of TYROBP regulating compounds

Drug-induced gene expression fold change was obtained from the Connectivity Map database [42], which consists of 6100 individual experiments, representing 1309 unique compounds. The 6100 individual expression profiles were merged into a single representative signature for the 1309 unique compounds, according to the prototype-ranked list method [28]. Each compound was scored according to the rank of *Tyrobp* expression fold change within its signature. Compounds were ranked in descending order of *Tyrobp* expression fold change and used for a secondary enrichment analysis of drug-target associations. For each compound in the drug signature library, referenced drug–target associations [45, 83] and predicted off-targeting [36, 37] were collected. For each of these features, we calculated a running sum enrichment score, reflecting whether that feature was over-represented among the compounds at the top (associated with *Tyrobp* upregulation) or at the bottom (associated with *Tyrobp* down-regulation). Two-tailed *p* values were based on comparison with 10,000 permuted null scores, generated from randomized drug target sets that contain an equivalent number of compounds to the true set under evaluation, and adjusted using the Benjamini–Hochberg method [6]. Computational screening and chemogenomic enrichment analysis were performed using the R project for statistical computing version 3.2.5 [62].

### Data and software availability

Gene expression data generated contributing to the described study will be deposited electronically to the Synapse Web Portal (https://www.synapse.org) in accordance with data sharing policies established by the NIH Accelerating Medicine Partnership (AMP) AD consortium. Specific software will also be made available upon request.

## Results

### TYROBP deficiency or absence does not modify the number and size of Aβ plaque depositions nor the number of microglial cells in prefrontal cortex and hippocampus of *APP/PSEN1* mice

**Fig. 1:**
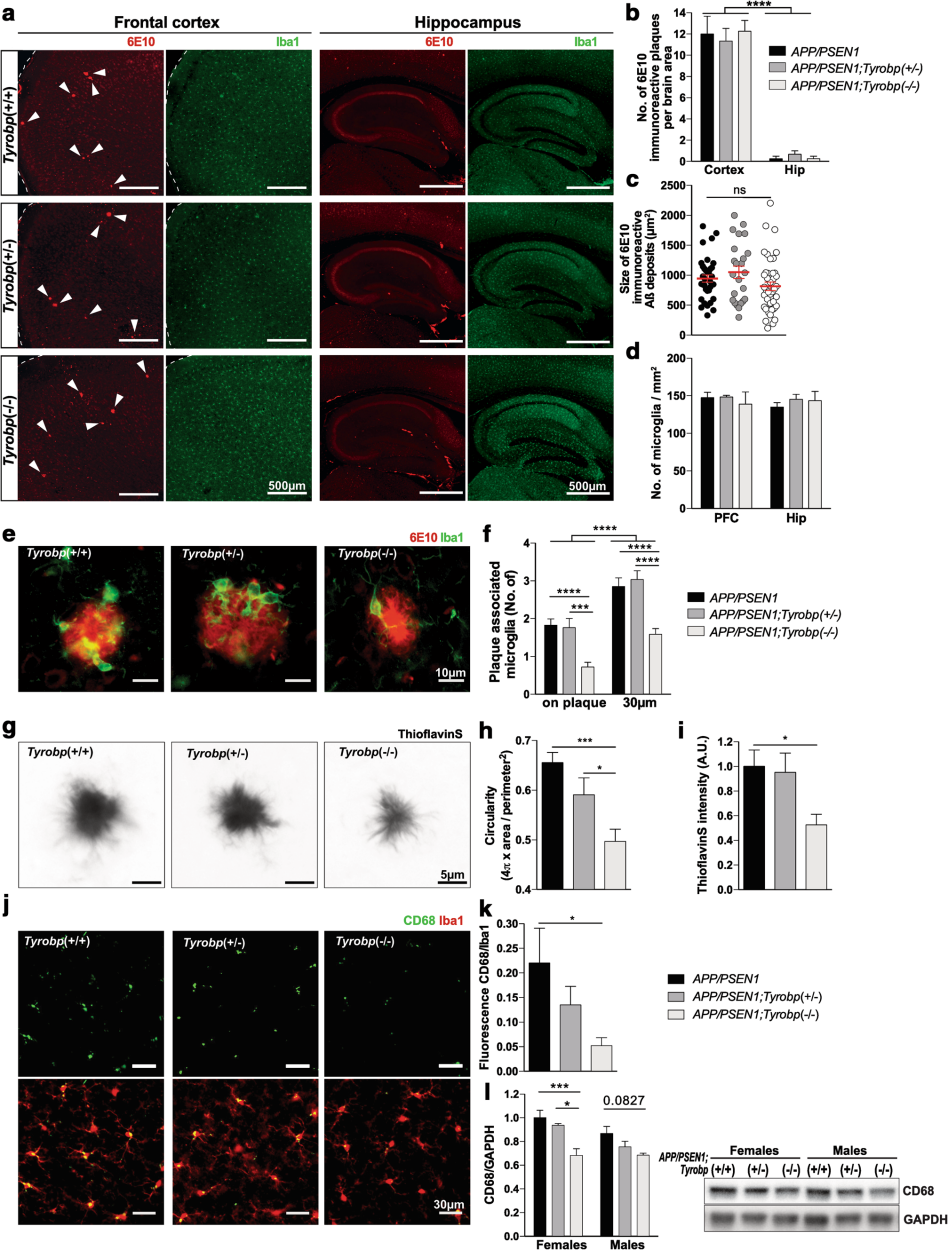
A decrease in TYROBP protein impairs Aβ deposits compaction, microglial activation, and recruitment around Aβ deposits in 4-month-old *APP/PSEN1* mice. **a** Images of Iba1-immunostained microglia (*green*) and 6E10-immunoreactive plaques (*red*) in frontal cortices and hippocampi of *APP/PSEN1, APP/PSEN1; Tyrobp* ^+*/*−^ and *APP/PSEN1; Tyrobp* ^−*/*−^ mice. Arrows indicate location of the plaques. *Scale bar* 500 µm. **b** Quantification of the number of 6E10-immunoreactive Aβ deposits in cortices and hippocampi (Hip) of *APP/PSEN1, APP/PSEN1; Tyrobp* ^+*/*−^ and *APP/PSEN1; Tyrobp* ^−*/*−^ mice. **c** Measurements of the size of 6E10-immunoreactive Aβ deposits in cortices of *APP/PSEN1* mice WT, deficient or null for *Tyrobp*. **d** Quantification of the number of Iba1-immunostained microglia in frontal cortices and hippocampi of *APP/PSEN1, APP/PSEN1; Tyrobp* ^+*/*−^ and *APP/PSEN1; Tyrobp* ^−*/*−^ mice. **e**, **f** Images of Iba1-immunostained microglia (*green*) and 6E10-immunoreactive plaques (*red*) and quantification of numbers of cortices plaque-associated microglia located on or within 30 µm radius of 6E10 immunoreactive Aβ plaques in *APP/PSEN1, APP/PSEN1; Tyrobp* ^+*/*−^ and *APP/PSEN1; Tyrobp* ^−*/*−^ mice. *n* = 3–4 mice per group. *Scale bar* 10 µm. **g**–**i** Images of thioflavin S-labeled amyloid plaques (**g**), circularity (**h**) and quantification of fluorescence intensity (**i**) of thioflavin S-labeled amyloid plaques from *APP/PSEN1, APP/PSEN1; Tyrobp* ^+*/*−^ and *APP/PSEN1; Tyrobp* ^−*/*−^ mice. *n* = 3–4 mice per group. *Scale bar* 5 µm. **j**, **k** Images of phagocytic microglial marker CD68 (*green*) and Iba1 (*red*) co-immunostaining (**j**) and quantification of fluorescence intensity of CD68 (**k**) in *APP/PSEN1, APP/PSEN1; Tyrobp* ^+*/*−^ and *APP/PSEN1; Tyrobp* ^−*/*−^ mice. *n* = 3–4 mice per group. *Scale bar* 30 µm. **i** Western blot analysis of CD68 in brain protein homogenates from *APP/PSEN1, APP/PSEN1; Tyrobp* ^+*/*−^ and *APP/PSEN1; Tyrobp* ^−*/*−^ mice. *n* = 3–6 mice per group. At least two independent western blot analyses were performed. Representative immunoreactive bands from the same western blot are shown on the right. One-way ANOVA corrected for multiple comparisons (Tukey) was used for (**c**, **h**, **i**, **k**) and Two-way ANOVA corrected for multiple comparisons (Tukey) was used for (**b**, **d**, **f**, **l**), *p < 0.05; ***p < 0.001; ****p < 0.0001. Data presented as mean ± SEM

We assessed whether TYROBP deficiency or absence modulates Aβ deposition in *APP/PSEN1* mice. We did not observe differences in number or size of 6E10 immunoreactive plaques in cortices or in the hippocampi of *APP/PSEN1* mice heterozygous or KO for *Tyrobp* as compared to *APP/PSEN1* mice with normal levels of TYROBP (Fig. 1a–c). It is important to note that 4-month-old *APP/PSEN1* mice represent an early time point of AD pathology, and all genotypes presented very little Aβ deposition in the hippocampus as compared to the cortex.

Reduction in total number of microglia has been observed in older TREM2 KO mice with Aβ pathology [30, 81], most likely due to a reduction of microglia proliferation. No differences were observed in younger mice. In our hands, Iba1 immunostaining in 4-month-old *APP/PSEN1* mice deficient or null for *Tyrobp* did not show differences in the total number of microglia in (pre)frontal cortices (PC) nor in hippocampi as compared to *APP/PSEN1* mice with normal levels of TYROBP (Fig. 1a, d). Similar results were observed in WT mice with normal or absent TYROBP (see Suppl. Figure 1).

### Loss of TYROBP reduces plaque compaction, microglia clustering, and phagocytosis

When 5XFAD mice, which develop rapid and aggressive amyloid pathology and neuronal loss [56], were rendered deficient or null for TYROBP or TREM2, microglial clustering around plaques and plaque compaction were reduced at 4 months of age [85]. We observed decreased microglial recruitment on and around antibody 6E10-immunoreactive Aβ deposits in the PC of 4-month-old *APP/PSEN1;Tyrobp* ^−*/*−^ mice as compared to *APP/PSEN1* mice with a normal level of TYROBP (Fig. 1e, f). We also observed reduced compaction and fluorescence intensity of thioflavin S reactive plaques (Fig. 1g–i).

We next assessed by immunostaining the level of a phagocytic marker CD68 [88] in the PC of *APP/PSEN1* mice WT, deficient or KO for TYROBP. In *APP/PSEN1;Tyrobp* ^−/−^ mice, we observed a decreased expression of CD68 in microglial Iba1-positive cells as compared to *APP/PSEN1* mice (Fig. 1j–k). *APP/PSEN1* mice heterozygous for *Tyrobp* did not present a statistically significant reduction of CD68 expression. Accordingly, the level of CD68 in hemibrain protein homogenates was lower in *APP/PSEN1;Tyrobp* ^−*/*−^ as compared with that observed in *APP/PSEN1* mice (Figure i, j). These data support an interpretation that microglial phagocytic activity was reduced in AD mice in the absence of TYROBP (Table 1 for a summary of results).

**Table 1:** Summary of the assays performed and results in *APP/PSEN1; Tyrobp* ^−*/*−^ vs. *APP/PSEN1*, *APP/PSEN1; Tyrobp* ^+*/*−^ vs. *APP/PSEN1* and *APP/PSEN1; Tyrobp* ^−*/*−^ vs. *APP/PSEN1; Tyrobp* ^+*/*−^

Assays	*APP/PSEN1; Tyrobp* (−/−) vs. *APP/PSEN1*		*APP/PSEN1; Tyrobp* (+/−) vs. *APP/PSEN1*		*APP/PSEN1;Tyrobp* (−/−) vs. *APP/PSEN1; Tyrobp * (+/−)	
	Females	Males	Females	Males	Females	Males
Aβ40						
TBS fraction	ns		ns		ns	
Triton-X fraction	ns		ns		↓	ns
Formic acid fraction	ns	↓	ns		ns	
Aβ42						
TBS fraction	ns		ns		ns	
Triton-X fraction	ns		ns		ns	
Formic acid fraction	ns		ns		ns	
Aβ42/40						
TBS fraction	ns		ns		ns	
Triton-X fraction	ns		ns		↑	ns
Formic acid fraction	ns		ns		ns	
NU-4	↓	↓ *p* = 0.08	ns	↓	ns	
A11	↓	ns	↓	ns	ns	
OC	↓ *p* = 0.06	ns	ns		ns	
CD68 (protein)	↓	↓ *p* = 0.06	ns		ns	
Phospho-TAU	↓	ns	ns		ns	
Synaptophysin (protein)	ns		ns		ns	
LAMP1 (protein)	↓	↓	↑	ns	↓	↓

*↓* Decreased level, *↑* increased level, *ns* not significant

### Aβ levels and oligomeric Aβ in *APP/PSEN1* mice deficient or null for TYROBP

We assessed whether TYROBP deficiency or absence modulates levels of Aβ species in *APP/PSEN1* mice. We measured levels of Aβ40 and Aβ42 in TBS, Triton-X, and formic acid-soluble Aβ fractions from brains of 4-month-old male and female *APP/PSEN1* mice on a *Tyrobp* heterozygous or null background (Suppl. Figure 2). In males, deletion of one or both *Tyrobp* alleles did not alter levels of Aβ40, Aβ42 or Aβ42/40 ratio in any of the three fractions as compared to male *APP/PSEN1* mice (Suppl. Figure 2a–i). Female *APP/PSEN1;Tyrobp* ^−*/*−^ mice exhibited lower levels of Aβ40 in Triton-X and formic acid fractions relative to *APP/PSEN1* mice, resulting in an increase in the Aβ42/40 ratio in the Triton-X fraction. This was not observed in the formic acid fraction (see Suppl. Figure 2a–i). Notably, female *APP/PSEN1* mice WT, heterozygous or knockout (KO) for *Tyrobp* had higher levels of Aβ40 and 42 in the Triton-X and formic acid fractions when compared to genotype-matched males.

**Fig. 2:**
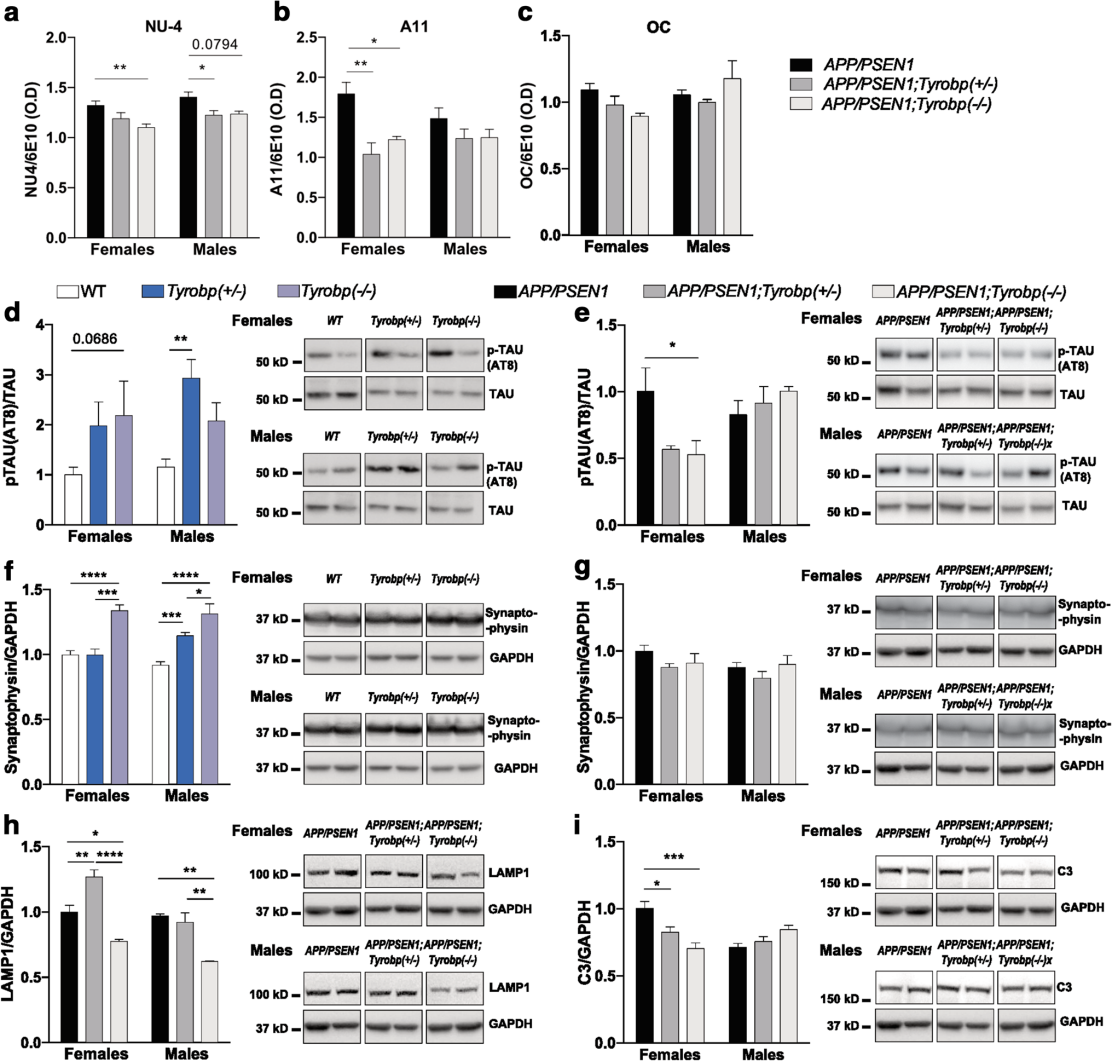
A decrease in TYROBP protein decreases oligomeric Aβ levels and alters phospho-TAU, synaptophysin, LAMP1, and complement C3 levels in 4-month-old *APP/PSEN1* mice. **a**–**c** Hemibrains of male and female *APP/PSEN1* (*n* = 4–6), *APP/PSEN1; Tyrobp* ^+*/*−^ (*n* = 3–8) and *APP/PSEN1; Tyrobp* ^−*/*−^ (*n* = 3–4) mice were processed via differential detergent solubilization to produce fractions of TBS soluble, Triton-X soluble, and formic acid soluble Aβ. Oligomeric Aβ was assessed from the TBS-soluble fraction via dot blot analyses using NU-4 (**a**), A11 (**b**) and OC (**c**) antibodies. **d**–**i** Western blot analysis in brain protein homogenates from 4-month-old male and female mice *WT, Tyrobp* ^+*/*−^, *Tyrobp* ^−*/*−^ and *APP/PSEN1, APP/PSEN1; Tyrobp* ^+*/*−^ and *APP/PSEN1; Tyrobp* ^−*/*−^ mice. **d**, **e** Phospho-tau (AT8 epitope)/total tau ratio for **d** *WT, Tyrobp* ^+*/*−^, *Tyrobp* ^−/−^ mice and **e** *APP/PSEN1, APP/PSEN1; Tyrobp* ^+*/*−^ and *APP/PSEN1; Tyrobp* ^−*/*−^ mice. **f**, **g** Synaptophysin level for **f** *WT, Tyrobp* ^+*/*−^, *Tyrobp* ^−/−^ mice and **g** *APP/PSEN1, APP/PSEN1; Tyrobp* ^+*/*−^ and *APP/PSEN1; Tyrobp* ^−*/*−^ mice. **h** Marker of dystrophic neurites (Lamp1) in *APP/PSEN1, APP/PSEN1; Tyrobp* ^+*/*−^ and *APP/PSEN1; Tyrobp* ^−*/*−^ mice. **i** Complement C3 in *APP/PSEN1, APP/PSEN1; Tyrobp* ^+*/*−^ and *APP/PSEN1; Tyrobp* ^−*/*−^ mice. At least two independent western blot analyses were performed. Representative immunoreactive bands from the same western blot are shown on the right. *n* = 3–6 mice per group. Two-way ANOVA corrected for multiple comparisons (Tukey) was used for all statistical comparisons in male and female mice, *p < 0.05; **p < 0.01; ***p < 0.001, ****p < 0.0001. Data presented as mean ± SEM

We next assayed oligomeric Aβ peptides using antibodies NU-4, A11, and OC antibodies to distinguish among various Aβ conformers (Fig. 2a–c). Higher levels of oligomeric Aβ reactive with these antibodies have been correlated with impaired cognitive performances in humans and mice [49]. We and others [40, 73] have reported an association of excess levels of NU4-epitope-containing oligomeric Aβ with deficits in learning behavior in AD mouse models. NU-4 reactive oligomer levels were reduced in both male and female *APP/PSEN1* mice with deficiencies in TYROBP as compared to levels observed in *APP/PSEN1* mice with normal TYROBP (Fig. 2a). A11 reactive oligomer levels were also reduced in female mice with reduced TYROBP as compared to *APP/PSEN1* mice WT for *Tyrobp* (Fig. 2b). TYROBP level played no obvious role in determining levels of OC epitope-containing oligomeric Aβ in this system (Fig. 2c). (See Table 1 for a summary of results).

### Phospho-TAU, synaptophysin, LAMP1, and C3 levels are altered in *APP/PSEN1* and WT mice with reduced or absent TYROBP

In addition to amyloid deposition, *APP/PSEN1* mice develop hyperphosphorylated microtubule-associated protein TAU (MAPT). We assayed the phosphorylation status of MAPT in WT (nontransgenic) and *APP/PSEN1* mice with normal, reduced, or absent TYROBP (Fig. 2d, e). We observed an apparent increased stoichiometry of TAU phosphorylation in male mice deficient for TYROBP as compared to WT mice (Fig. 2d). Females deficient for TYROBP demonstrated a trend toward increased phosphorylation of TAU as compared to WT mice (*p* = 0.07). In the presence of mutant *APP/PSEN1* transgenes, there was a reduction in the stoichiometry of TAU phosphorylation in female mice with reduced or absent TYROBP, but no difference in male mice (Fig. 2e).

To examine synaptic integrity, we measured the levels of the presynaptic neuronal marker, synaptophysin (Fig. 2f, g). Synaptophysin was increased in male and female *Tyrobp* ^−*/*−^ mice as compared to WT mice (Fig. 2f), but no difference was observed between groups in *APP/PSEN1* mice heterozygous-null or homozygous-null for *Tyrobp* (Fig. 2g). Notably, however, LAMP1, a lysosomal protein enriched in dystrophic neurites [15, 21], was decreased in both male and female *APP/PSEN1; Tyrobp* ^−*/*−^ mice relative to *APP/PSEN1* alone (Fig. 2h). Excessive activation of the complement system is an early event in AD leading to synapse loss. The level of complement C3 was decreased in female *APP/PSEN1* homozygous-null for *Tyrobp* relative to those expressing *APP/PSEN1* alone (Fig. 2i). No difference was observed in corresponding male mice (see Table 1 for a summary of results). Despite the relatively minor effect size, likely due to the early stage of AD pathology in 4-month-old *APP/PSEN1* mice, when taken together, these results are consistent with a conclusion that decreased expression of *Tyrobp* may have beneficial effects in the proteinopathy of AD.

### Electrophysiological changes in *APP/PSEN1* mice deficient for TYROBP

**Fig. 3:**
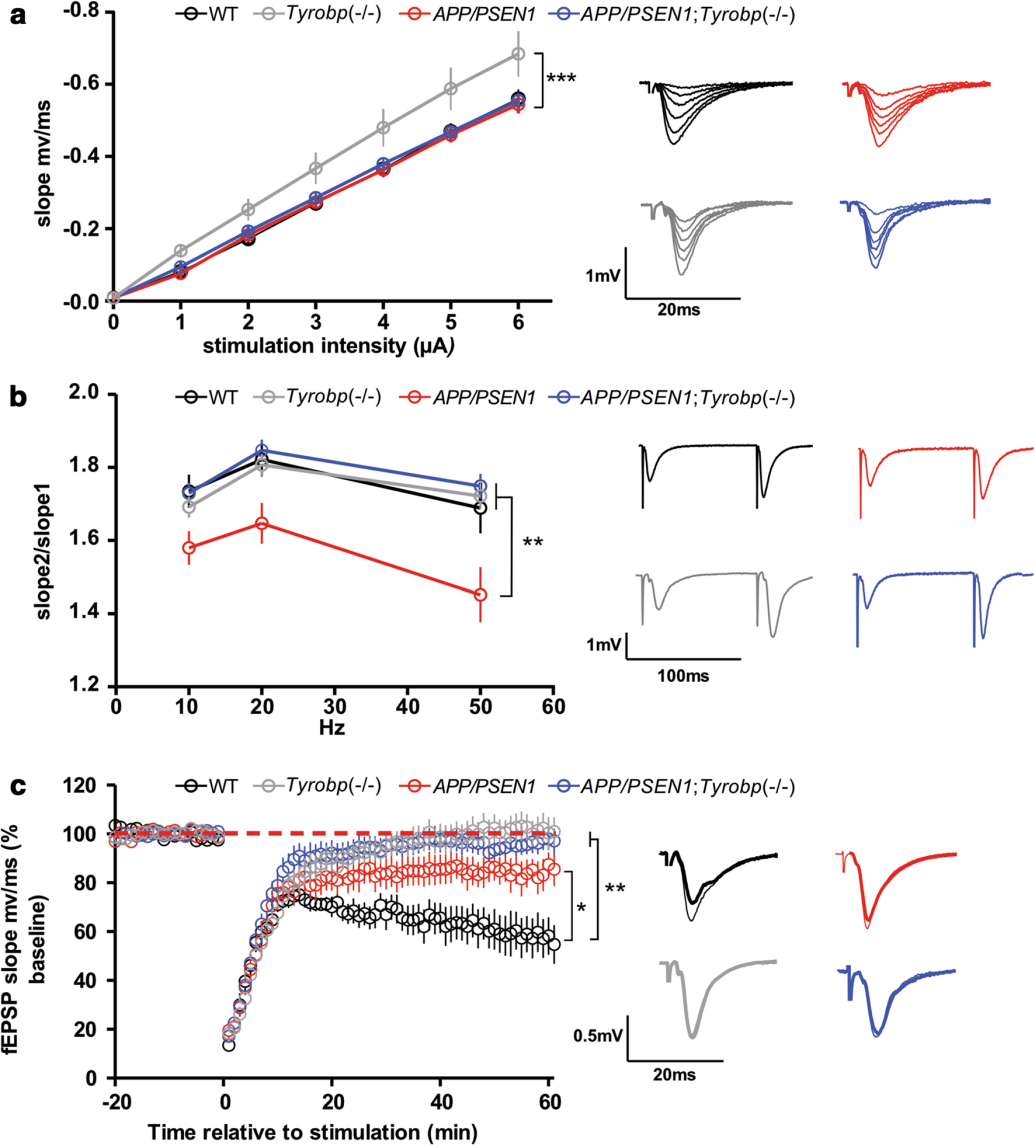
A decrease in TYROBP protein alters excitatory synaptic transmission in the hippocampus in 4-month-old *APP/PSEN1* mice and interacts with the *APP/PSEN1* genotype. In all panels, summary graphs are shown on the *left* and representative traces on the *right*. **a** Basal synaptic function is increased in *Tyrobp* ^−/−^ mice, but is unaffected by other transgenic genotypes. The slope of the input/output relationship was steeper for the *Tyrobp* ^−/−^ mice than for all other genotypes (*p* < 0.05), which did not differ among themselves. **b** *APP/PSEN1* mice showed reduced paired-pulse facilitation (PPF) relative to other genotypes, which did not differ among themselves. **c** Synaptically induced long-term depression (LTD) was impaired in all recombinant mice. Analysis over the final 5 min of the recordings showed the most profound deficits for *Tyrobp* ^−*/*−^ and *APP/PSEN1;Tyrobp* ^−*/*−^ mice, both of which were significantly less depressed than in *APP/PSEN1* mice. Wild-type (WT) controls showed significantly greater depression than the other genotypes. One-way ANOVA corrected for multiple comparisons (Tukey) was used for statistical comparisons, *p < 0.05, **p < 0.01; ***p < 0.001. Data presented as mean as mean ± SEM

TYROBP, amyloid, and presenilin proteins play important roles in excitatory synaptic transmission at Shaffer collateral-CA1 pyramidal cell synapses [52, 68]. All of the recombinant mouse models tested in this report showed either altered basal synaptic function, reduced plasticity, or both. Basal synaptic efficiency, as measured by the slope of the input–output relationship, was normal in *APP/PSEN1* and *APP/PSEN1; Tyrobp* ^−*/*−^ as compared to WT mice (Fig. 3a). Interestingly, the slope of the input–output relationship was increased in *Tyrobp* ^−/−^ mice compared to WT, *APP/PSEN1*, and *APP/PSEN1; Tyrobp* ^−*/*−^ suggesting an increased basal synaptic activity in absence of TYROBP (Fig. 3a).

We tested the possibly that this effect of *Tyrobp* deletion was presynaptically mediated using paired-pulse facilitation (PPF), a short-term form of synaptic plasticity that is sensitive to the probability of transmitter release [9]. PPF was normal in *Tyrobp* ^−/−^ mice (Fig. 3b) suggesting that the increase in basal efficiency observed in *Tyrobp* ^−/−^ mice most likely reflects postsynaptic regulation such as increased expression and/or function of synaptic AMPA-type glutamate receptors (AMPARs). These data also raise the possibility of impaired endocytosis of AMPARs [87]. In contrast, PPF was depressed in *APP/PSEN1* mice relative to WT controls, indicating an increase in transmitter release probability. Importantly, *Tyrobp* deletion reversed the deleterious effect of *APP/PSEN1* on presynaptic function, since PPF was normal in slices from *APP/PSEN1; Tyrobp* ^−*/*−^ mice.

We also examined the effects of the different *APP/PSEN1* and *Tyrobp* genotypes on long-term depression (LTD), a persistent form of plasticity whose expression depends on endocytosis of postsynaptic AMPARs [38]. For these experiments, we used a synaptic induction protocol that induces a prominent protein synthesis-dependent “late” phase of LTD [69]. Slices from the *APP/PSEN1* mice showed impaired LTD (Fig. 3c), similar to that reported in older *APP/PSEN1* mice. Similar results were observed following a weaker induction protocol [13] or when late LTD was induced by metabotropic glutamate receptor activation (mGluR-LTD) [84]. LTD was even more impaired in *Tyrobp* ^−/−^ and *APP/PSEN1; Tyrobp* ^−*/*−^ mice. Thus, unlike the phenotypes for basal efficiency and PPF, superimposition of TYROBP deficiency on the *APP/PSEN1* mutations failed to restore normal LTD.

### Barnes maze

**Fig. 4:**
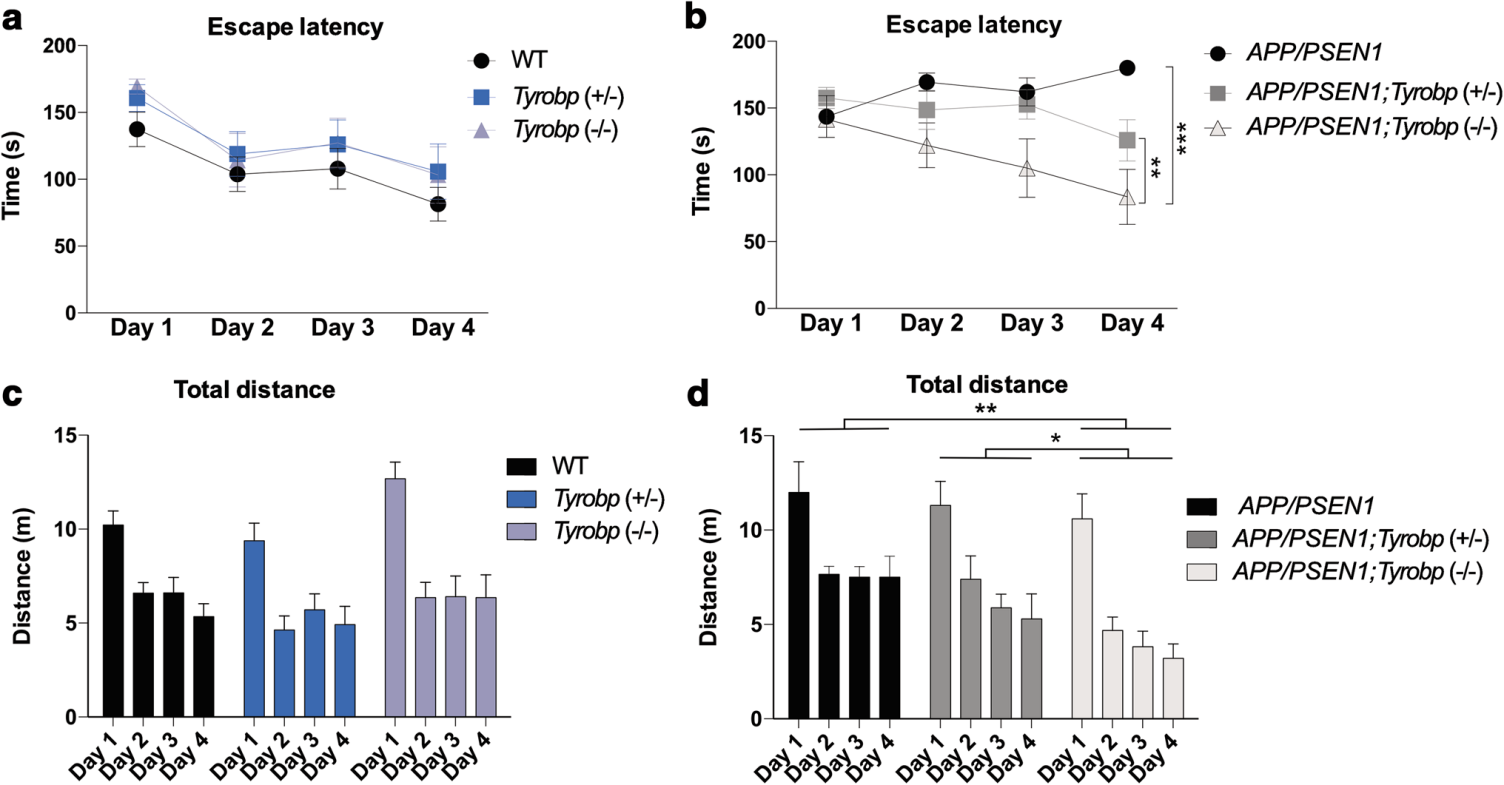
A decrease in TYROBP protein improves spatial learning and memory in the Barnes Maze Test in 4-month-old *APP/PSEN1* mice. **a**–**d** 6 groups of 4-month-old mice were used: wild-type (WT) (*n* = 14), *Tyrobp* ^+*/*−^ (*n* = 9), *Tyrobp* ^−/−^ (*n* = 10), *APP/PSEN1* (*n* = 5), *APP/PSEN1; Tyrobp* ^+*/*−^ (*n* = 9) or *APP/PSEN1; Tyrobp* ^−*/*−^ (*n* = 11). **a**, **b** Mean latencies to enter the target hole for **a** *APP/PSEN1* negative mice and **b** *APP/PSEN1* positive mice. **c**, **d** Mean distances traveled for **c** *APP/PSEN1* negative mice and **d** *APP/PSEN1* positive mice. Two-way ANOVA corrected for multiple comparisons (Tukey) was used for all statistical comparisons, *p < 0.05; **p < 0.01; ***p < 0.001. Data presented as mean ± SEM

We next probed the effect of *Tyrobp* deletion on the modulation of spatial learning and memory using the Barnes Maze Test (Fig. 4a–d). The escape latency and distance traveled of *Tyrobp* heterozygous- and homozygous-null mice were identical to WT littermates (Fig. 4a, b). In the presence of *APP/PSEN1* mutations, deficiency of TYROBP improved learning and memory relative to *APP/PSEN1* with normal TYROBP levels (Fig. 4c, d). This improvement was associated with a reduction in the time spent finding the hidden location (target hole) and a smaller distance traveled in all quadrants. These behavioral data are consistent with a beneficial effect of the *Tyrobp* deletion on the *APP/PSEN1* phenotype.

### Differential gene expression analysis of *Tyrobp*^−/−^ and *Tyrobp*^+/−^ mice relative to WT mice

**Fig. 5:**
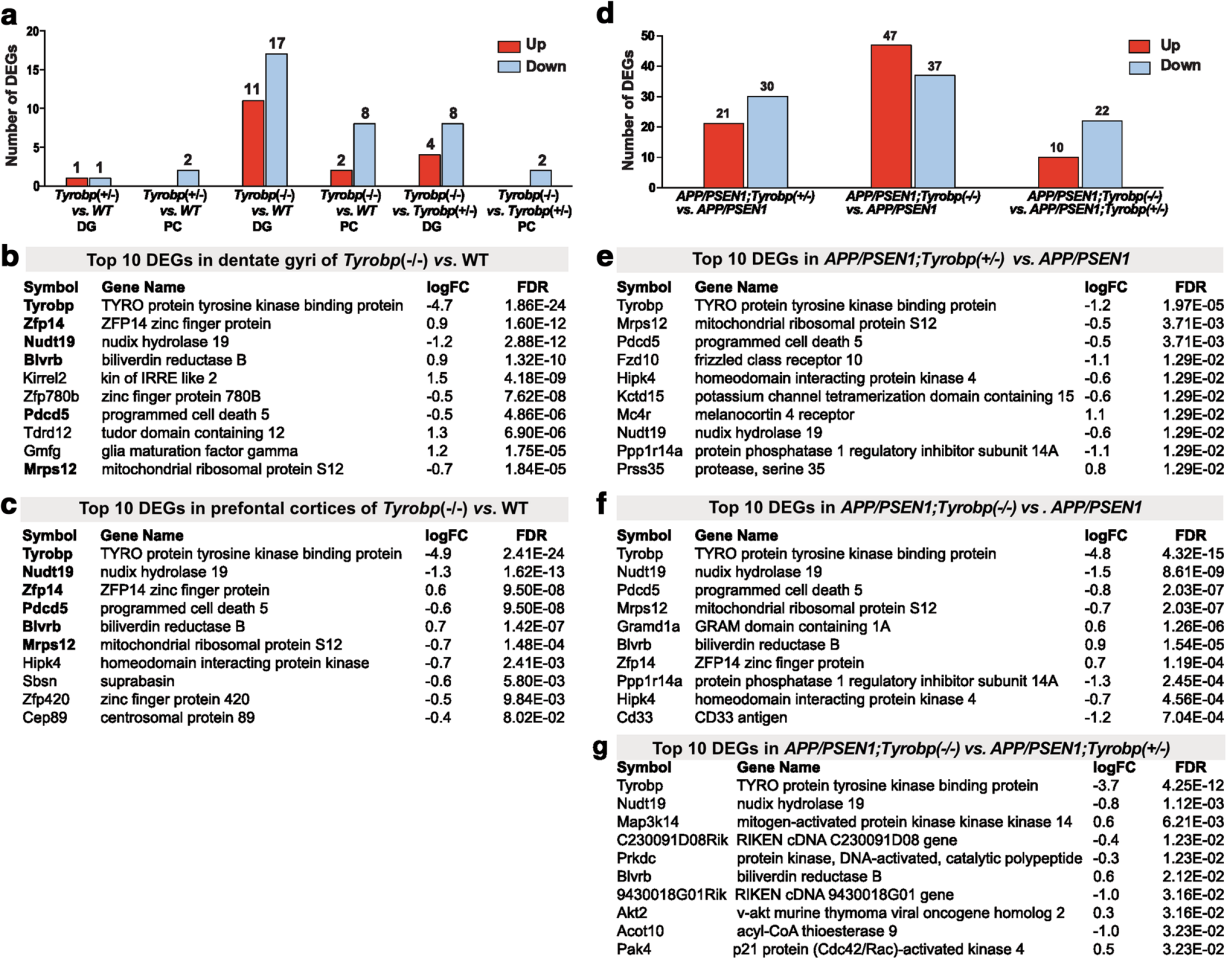
Differential gene expression analysis suggests potential molecular mechanisms associated with TYROBP deficiency. **a**–**c** Differential gene expression analysis in dentate gyrus and prefrontal cortex of *Tyrobp* ^−*/*−^ *, Tyrobp* ^+*/*−^ and *WT* mice. **a** Number of up- and down-regulated genes in *Tyrobp* ^−/−^ and *Tyrobp* ^+/−^ vs. *WT* and *Tyrobp* ^−/−^ vs. *Tyrobp* ^+/−^. **b** Top 10 differentially expressed genes in **b** dentate gyrus of *Tyrobp* ^−/−^ vs. *WT* and **c** prefrontal cortex of *Tyrobp* ^−/−^ vs. *WT.* Bolding highlights differentially expressed genes shared across dentate gyrus and prefontal cortex. RNA sequencing was performed on a total of 47 samples (*Tyrobp* ^−/−^ *n* = 8 males and 8 females, *Tyrobp* ^+/−^ *n* = 8 males and 8 females, and WT *n* = 7–8 samples, eight males and eight females, for each brain regions). All analyses were corrected for sex effect. Differential gene expression threshold was set at fold change ≥1.2 and adjusted *p* value <0.1. *DG* dentate gyrus, *PC* prefrontal cortex. **d**–**g** Differential gene expression analysis in prefrontal cortex of *APP/PSEN1;Tyrobp* ^−*/*−^ *, APP/PSEN1; Tyrobp* ^+*/*−^ and *APP/PSEN1* mice at 4-months-old. **d** Number of up- and down-regulated genes in *APP/PSEN1;Tyrobp* ^−*/*−^ and *APP/PSEN1;Tyrobp* ^+*/*−^ vs. *APP/PSEN1* and *APP/PSEN1; Tyrobp* ^−*/*−^ vs. *APP/PSEN1; Tyrobp* ^+*/*−^. **e** Top 10 differentially expressed genes in *APP/PSEN1; Tyrobp* ^+*/*−^vs. *APP/PSEN1;* **f** *APP/PSEN1; Tyrobp* ^−*/*−^ vs. *APP/PSEN1 *and **g** *APP/PSEN1; Tyrobp* ^−*/*−^ vs. *APP/PSEN1; Tyrobp* ^+*/*−^. RNA sequencing was performed on a total of 23 samples comprising of both male and female mice (*n* = 7–8 samples per genotype). All analyses were corrected for sex effect. Differential gene expression threshold was set at fold change ≥1.2 and false discovery rate (FDR) <0.1. (See Suppl. 3 and 4 for full list of differentially expressed genes)

Given our extensive database on the regional and disease-stage-specific transcriptomic changes in human LOAD [64, 79, 86], we began by investigating how *Tyrobp* deletion perturbed brain regional transcriptomes. We generated transcriptomic profiles from (pre)frontal cortices (PC) and dentate gyri (DG) (n = 24) for 4-month-old female and male *Tyrobp* ^−/−^ and *Tyrobp* ^+/−^ mice and compared with WT mice, including sex as a variable. In comparison with non-recombinant (WT) mice, we identified 10 differentially expressed genes (DEG) in the PC, and 28 DEG in the DG of *Tyrobp* ^−/−^ mice [false discovery rate (FDR) <0.1] (Fig. 5a). We also identified 2 DEG in the PC and DG of *Tyrobp* ^+/−^ mice vs. WT mice. *Tyrobp* was the top DEG among the different models and brain areas (logFC = −1.1 and −4.8 in *Tyrobp* ^+/−^ and *Tyrobp* ^−/−^, respectively) (Fig. 5b, c). Overall, we observed strong overlap of the DEG between the different brain areas in mice KO for *Tyrobp* (Fig. 5b, c for top 10 DEG in PC and DG, see Suppl. 3 for full DEG results). Thus, eight out of ten DEG in the PC of *Tyrobp* ^−/−^ were also differentially expressed in the DG of *Tyrobp* ^−/−^. Among them are two genes that have been proposed as early biomarkers of AD: biliverdin reductase B (*Blvrb*) (logFC = 0.9) [54] and Nudix motif 19 (*Nudt19)* (logFC −1.2) [1]. We also noted a strong trend toward down-regulation of *Cd33* in the DG of *Tyrobp* ^−*/*−^ mice (log FC = −0.9, FDR = 0.077). Recent genome-wide studies identified *CD33* as a late-onset AD susceptibility variant [24, 55]. Moreover, CD33 protein is elevated in AD brain and has been associated with amyloid pathology and disease progression [10, 22, 78]. As expected with such small DEG signatures, we did not observe significantly dysregulated Gene Ontology (GO) term enrichments in *Tyrobp* heterozygous- or homozygous-null mice using DAVID, Ingenuity Pathway Analysis (IPA) or gene set enrichment analysis (GSEA).

### Differential gene expression and enrichment analysis of *APP/PSEN1;Tyrobp*^−*/*−^, *APP/PSEN1;Tyrobp*^+*/*−^ and *APP/PSEN1* mice

We generated transcriptomic profiles from 23 PC samples from 4-month-old male and female *APP/PSEN1* mice that were either heterozygous- or homozygous-null, or WT for *Tyrobp*. Sex effect was taken into account for all analyses. In comparison to *APP/PSEN1* mice, we identified 84 DEG in *APP/PSEN1;Tyrobp* ^−*/*−^ and 51 DEG in *APP/PSEN1; Tyrobp* ^+*/*−^ (FDR <0.1) (Fig. 5d–g. See Suppl.4 for full DEG results). All of the ten DEG detected in the PC of *Tyrobp* ^−*/*−^ *vs*. WT mice were also differentially expressed in the PC of *APP/PSEN1; Tyrobp* ^−*/*−^ vs. *APP/PSEN1* comparison. The increased signature size in *Tyrobp* ^−*/*−^ in the *APP/PSEN1* background provides strong independent support for the conclusion that TYROBP is relevant not only in human AD [86] but also in the amyloid-depositing mouse brain AD model.

As above, *Tyrobp* was the top DEG in both *APP/PSEN1* deficient and KO for *Tyrobp* (logFC = −1.2 and −4.8, respectively). Comparison of *APP/PSEN1; Tyrobp* ^−*/*−^ *vs*. *APP/PSEN1; Tyrobp* ^+*/*−^ highlighted 32 DEG. Interestingly, we found evidence in *APP/PSEN1* mice that were heterozygous- or homozygous-null for *Tyrobp* for several DEG associated with AD and/or memory loss. TYROBP deficiency produced changes in *Cd33* expression providing independent confirmation of similar phenomena observed by others using different approaches [12]. Also, *Sirt2* expression was increased in *APP/PSEN1; Tyrobp* ^−*/*−^ (logFC = 0.4). A *SIRT2* polymorphism has been associated with increased LOAD susceptibility [82] and its level of expression is linked with neurodegenerative disease, likely due to its role in lysosome-mediated authophagic turnover [18, 51, 58]. *Igfbp2* expression was decreased in *APP/PSEN1; Tyrobp* ^+*/*−^ (logFC = −0.6). Several proteomic studies aiming to identify AD markers in human sera have reported an increased level of IGFBP2 in AD patients [39, 57]. Moreover, Pedrós et al. have shown an increased expression level of IGFBP2 in hippocampi of an *APP/PSEN1* mouse model similar to that which we used [60]. These data suggest that *Tyrobp*-related modulation of the expression of several AD-related genes only appears in the presence of cerebral amyloidosis and/or *APP/PSEN1* mutations.

**Fig. 6:**
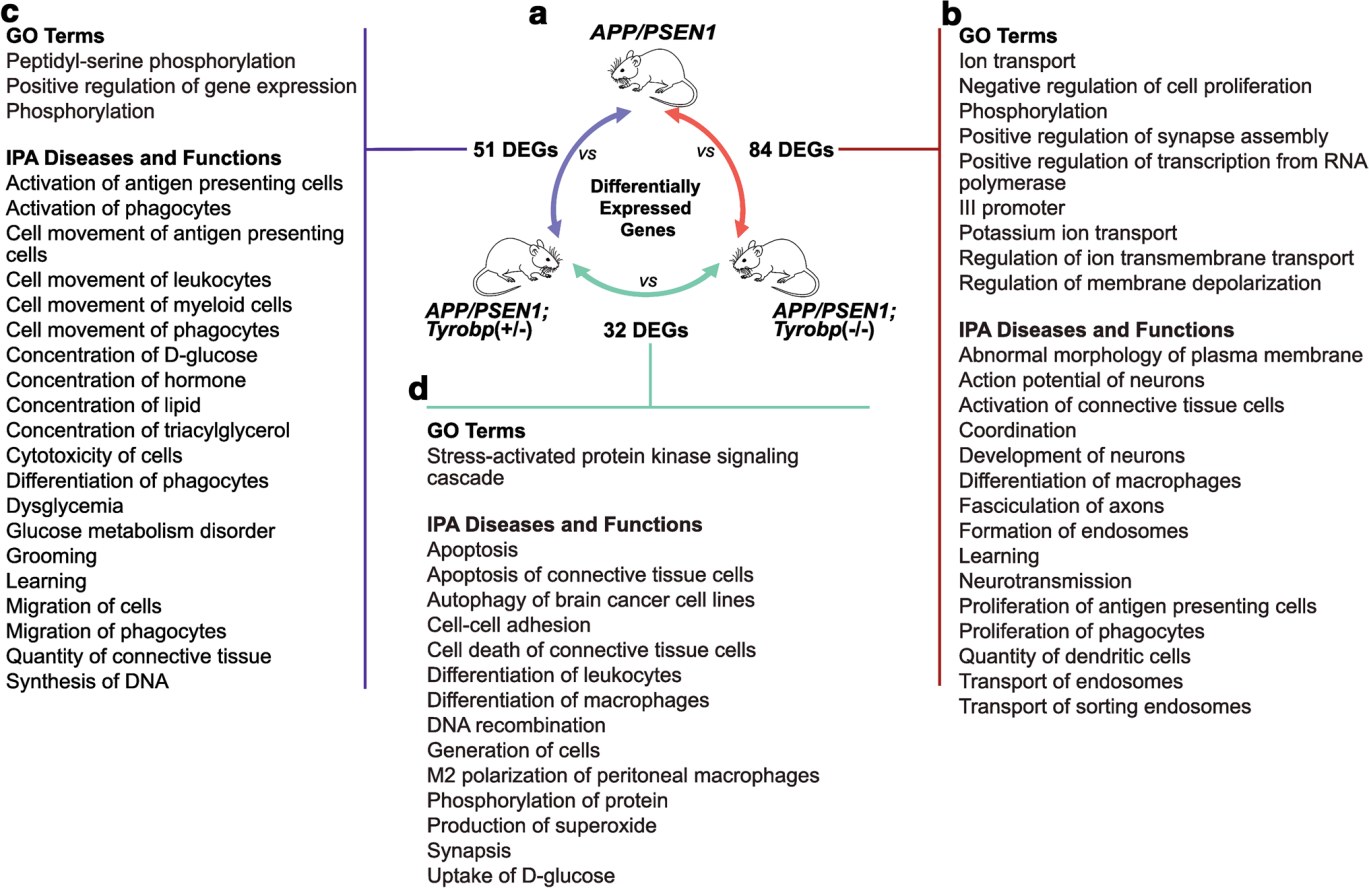
Gene enrichment analysis summary for prefrontal cortex of 4-month-old *APP/PSEN1;Tyrobp* ^−*/*−^ *, APP/PSEN1; Tyrobp* ^+*/*−^ and *APP/PSEN1* mice at suggests potential molecular mechanisms associated with TYROBP deficiency. **a** Schematic overview of comparisons between mouse groups. **b** Enrichment analysis and selected GO terms (DAVID) and diseases and functions (Ingenuity Pathway Analysis) in *APP/PSEN1; Tyrobp* ^−*/*−^ vs. *APP/PSEN1*, **c** in *APP/PSEN1; Tyrobp* ^+*/*−^ vs. *APP/PSEN1*, **d** in *APP/PSEN1; Tyrobp* ^−*/*−^ vs. in *APP/PSEN1; Tyrobp* ^+*/*−^. Enrichments shown were selected for known or suspected relevance to AD pathophysiology. Gene set enrichment threshold was set at *p* value <0.05. (See Suppl. 5 for full list of enrichments)

To identify biological pathways that may be dysregulated, we performed GSEA using DAVID and IPA (Fig. 6a). Comparisons between *APP/PSEN1* KO for *Tyrobp* and WT for *Tyrobp* highlighted common themes between DAVID and IPA analyses for perturbation of neurotransmission and ion transport (Fig. 6b and Suppl.5). These included potassium transport, general regulation of transmembrane ion transport, and depolarization and action potential of neurons. Other overlapping themes included neuronal, axonal fasciculation, and synapse assembly. Enrichment analysis with IPA comparing *APP/PSEN1; Tyrobp* ^+*/*−^ against *APP/PSEN1* detected dysregulation of immune function, including migration, movement, and activation of immune and phagocyte cells (Fig. 6c). Perturbation of metabolic functions was also detected. Using DAVID analysis, we noted a pervasive perturbation of protein phosphorylation signal transduction in *APP/PSEN1* mice either heterozygous- or homozygous-null for *Tyrobp* in comparison to *APP/PSEN1* mice on a *Tyrobp* wild-type background. This is potentially relevant to the role of TYROBP as a phosphotyrosine-signal-based sensor of extracellular debris and instigator and/or organizer of phagocytosis. This is also interesting in light of the evidence that Aβ cerebral amyloidosis in humans and mice is accompanied by hyperphosphorylation of cytoskeletal proteins. Comparisons between *APP/PSEN1; Tyrobp* ^−*/*−^ and *APP/PSEN1; Tyrobp* ^+*/*−^ showed a unique GO term involving stress-activated protein kinase signaling cascade (Fig. 6d). Pathways identified by IPA included production of superoxide, apoptosis, and differentiation/polarization of macrophages. Excessive production of superoxide can induce an uncontrolled oxidative stress leading to increased microglia activation and neuronal apoptosis [80]. Oxidative stress may also promote production and deposition of Aβ and formation of neurofibrillary tangles [8, 14, 20, 46, 53].

### Gene regulatory network analysis

Gene regulatory network analysis is a powerful tool in identifying gene modules pathologically related to complex human diseases including AD [86]. To test if the DE signatures detected in the present study were enriched for any AD networks, we collected the co-expression network modules from our two AD cohorts and overlaid the DEG onto the co-expression network modules. We had previously constructed transcriptome-wide gene co-expression networks in different brain regions of postmortem samples from two AD cohorts, the Mount Sinai Brain Bank (MSBB) AD cohort [79] and the Harvard Brain Bank (HBB) AD cohort [86]. Importantly, the age of the mice in this study corresponds to an early stage of AD while our human postmortem co-expression networks from HBB correspond to later disease stages. Although we did not observe enrichments in *APP/PSEN1;Tyrobp* ^−*/*−^, we found that the DEG down-regulated in PC of *APP/PSEN1;Tyrobp* ^+*/*−^ (FDR <0.2) were enriched for several sub-networks from both the MSBB and the HBB AD cohorts, including insulin-like growth factor binding, skeletal development, immune system process, anion transport, and particularly the extracellular matrix (see Suppl.6). The up-regulated genes in *APP/PSEN1;Tyrobp* ^+*/*−^ mice showed enrichment for nucleobase nucleoside and nucleotide metabolic process sub-network (see Suppl. 6).

### Drug repurposing

Through the experiments described above, we identified benefits of TYROBP deficiency on multiple aspects of the phenotype in *APP/PSEN1* mice. When those AD model mice were also deficient in TYROBP, beneficial effects in gene expression, phosphorylation of tau, nerve terminal integrity, behavior, and electrophysiology were observed. The data indicate that reduction of *Tyrobp* gene expression could represent a novel computation- and mutation-based, immune-inflammatory therapeutic opportunity for treating or preventing LOAD. Therefore, we probed a comprehensive pharmacopeia database to determine whether safe existing medications would be predicted to reduce levels of *Tyrobp* mRNA or protein.

**Fig. 7:**
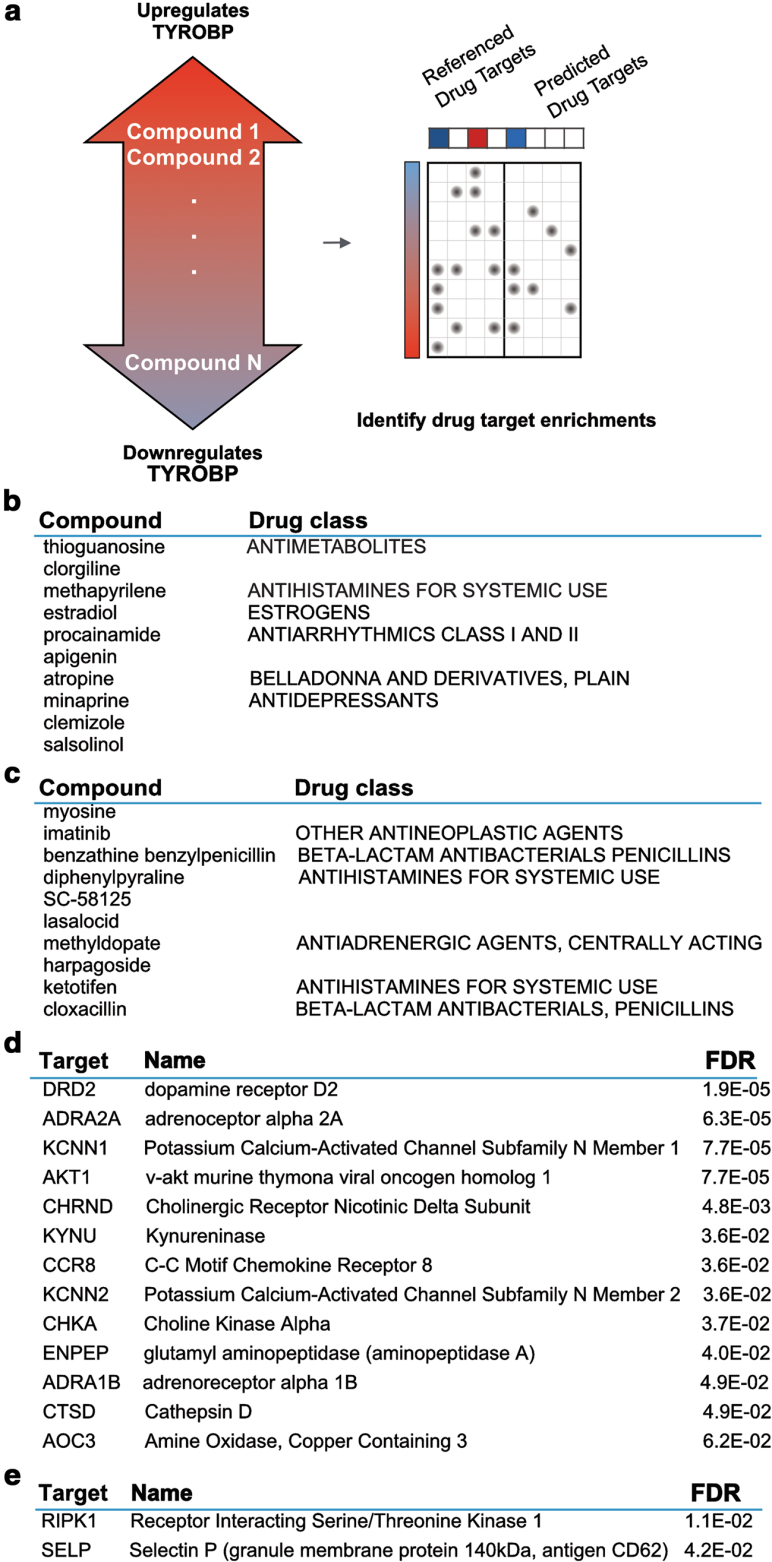
Computational analysis of a current pharmacopeia database identified compounds that would be predicted to cause up- or down-regulation of *TYROBP* expression. **a** Compounds were scored and ranked according to their associated *TYROBP* expression fold change, and then used as the basis for a secondary enrichment analysis to identify drug targets that associate with up- or down-regulation of *TYROBP*. Top 10 compounds that **b** up-regulate and **c** down-regulate *TYROBP* are shown. Drug targets enriched among compounds that **d** up-regulate, and **e** down-regulate *TYROBP* are shown

To identify small molecule compounds capable of perturbing *Tyrobp* expression (Fig. 7), we performed a computational screen against a library of drug-induced transcriptional profiles from Connectivity Map [42]. We scored 1309 unique compounds according to the rank of *Tyrobp* expression fold change (based on comparison to within-batch vehicle control assays). Top compounds associated with *Tyrobp* up and down regulation are summarized in Fig. 7b.

To explore the pharmacological context of these compounds, we performed a secondary enrichment analysis to identify drug targets that are associated with *Tyrobp* regulation (Fig. 7a, c). We found that compounds that regulate *Tyrobp* expression are enriched for multiple drug targets, including many with known links to Alzheimer’s pathology. These include LOAD risk-associated gene Cathepsin D (FDR = 4.9E−02) [16, 70, 72], and *Akt1* (FDR = 6.8E−04), a molecule that is activated in LOAD [65] and is associated with LOAD risk in Chinese Han AD patients with type 2 diabetes [50].

Of the targets enriched among compounds predicted to suppress *Tyrobp* expression (and so potentially representing therapeutic candidates), RIPK1 (FDR = 1.1E−02) was most strongly implicated. Interestingly, RIPK1, a key constituent of the necrosome, was recently shown in an independent study by other investigators to regulate context-dependent regulation of programmed necrosis via formation of an amyloid signaling complex [47]. Experimental validation of these predicted repurposable drugs is underway and will be reported in detail in a future publication.

## Discussion

Association of *TYROBP* with LOAD arose via a multiscale computational network approach [86]. The physical interaction between TREM2 and TYROBP as well as with other LOAD risk factors such as CR3, and SIRPβ1 [5, 7, 23, 55, 86] provided an important lead for our experimental strategy aiming to validate the important role of *TYROBP* in the pathogenesis of LOAD. We have previously defined via a multiscale computational network approach *TYROBP* as a strong candidate for playing the role of a key “hub” or “driver” gene in LOAD [86]. It is worth noting that two independent groups have also identified *TYROBP* as a driver of LOAD despite having followed different and highly idiosyncratic computational strategies [12, 48]. *CD33* is a known AD risk gene and a component of the *TYROBP* network. The regulation of *Cd33* by TYROBP reported herein as well as the regulation of *TREM2* by CD33 reported by de Jager and colleagues [12] provide compelling evidence in support of the role of *TYROBP* as a “driver” gene in LOAD. Capping off the evidence associating *TYROBP* with LOAD is the recent discovery that missense mutations in the coding region of the *TYROBP* gene are associated with AD risk [61]. Interestingly, in the same study, in vitro overexpression of the candidate pathogenic p.D50_L51ins14 *TYROBP* variant led to a strong reduction of TREM2 expression [61]. We have previously shown that *TYROBP* expression is elevated in AD brain and mouse models [64, 86], but it was not immediately apparent whether that elevation represented a pre-existing, predisposing factor or was a secondary reaction to LOAD pathology. Based on the data presented above, a *Tyrobp* null mutation appears to exert effects that would be characterized as beneficial with respect to both the normal physiology of neurons and the proteinopathy of LOAD.

The effects of *Tyrobp* knockdown or knockout on Aβ levels and Aβ oligomer conformers as defined by epitope content were limited to the reduction of the level of NU-4 and A11 type oligomers in TYROBP deficient *APP/PSEN1* mice. There were no consistent statistically significant differences on levels of total Aβ, Aβ40, Aβ42, or on levels of OC type Aβ oligomers. The relatively minor effect size notwithstanding, it is worth noting that the converging evidence from several laboratories (including our own) is that the NU-4 epitope is the signature of the Aβ oligomer strain that is most consistently neurotoxic [40, 73]. A11 and OC oligomer strains are not consistently neurotoxic. As reported above and in one of our previous studies [41], we noted sex differences in Aβ and oligomer levels suggesting an earlier progression of the disease in female than male *APP/PSEN1* mice.

The difference in Aβ levels observed between the male and female mice is of importance considering the sexual dimorphism observed in the phosphorylation status of TAU in *APP/PSEN1* background but not in WT background. Thus, the effect of a decreased *Tyrobp* expression on the stoichiometry of TAU phosphorylation appeared to be different in the presence or absence of *APP/PSEN1* mutations leading to amyloid deposition. Indeed, TYROBP deficiency tends to increase the phosphorylation of TAU on a WT background, but, on a *APP/PSEN1* background, loss of TYROBP decreased the phosphorylation status of TAU in female mice in the setting of higher Aβ loads as compared with males. Although the mechanism(s) by which microglia exert their effects on neuronal tau pathology remains unclear, several reports have linked TREM2 expression and hyperphosphorylated TAU [31, 33, 35]. These reports suggest that TREM2 deficiency could increase tauopathy in human tau-expressing models but could decrease tau pathology in AD mouse models displaying cerebral amyloidosis. Herein we report that a decreased expression of *Tyrobp* can have beneficial effects on tau pathology and neuronal injury in *APP/PSEN1* mouse model of AD. In accordance with our data, Strittmatter and colleagues [75] recently reported that mouse deficient for *Progranulin* presented an overexpression of *Tyrobp* network genes correlating with an increased neuronal injury and tau pathology in the absence of amyloid pathology [75].

As mentioned above, no differences were noted in number and size of Aβ plaque depositions and the general histological impact of TYROBP deficiency on plaque morphology and microglia recruitment was identical in appearance to that reported by Colonna and colleagues in their studies of TREM2*-*deficient mice [85]. Indeed, *Tyrobp* KO mice presented fewer microglia decorating each amyloid plaque without modification in the total number of microglia, and plaques exhibited less compact morphology. However, unlike the Colonna report wherein the reduced numbers of microglia per plaque were predicted to be associated with *increased severity* of the phenotype [85], we observed that this histological appearance was instead associated with *beneficial effects* on neuritic dystrophy, TAU metabolism, learning behavior, and neuronal electrophysiology. Although beyond the scope of this study, it will be interesting to determine whether overexpression leads to opposite results. In addition, recent papers from Lamb and colleagues [30], Yu and colleagues [32, 34], and Raha-Chowdhury and colleagues [63] raise the possibility that there could be aging-related and/or disease-stage-related changes in the effect of TYROBP. These papers focused on TREM2 and suggest that reduced TREM2 may be beneficial early in life (~4 months) while reduced TREM2 late in life (~8 months) could be detrimental. We are in the process of assessing whether a similar phenomenon occurs with TYROBP.

Electrophysiological assays revealed that the loss of TYROBP normalized some of the synaptic dysfunctions caused by the *APP/PSEN1* mutations. The strong increase in basal synaptic efficiency seen in the *Tyrobp* ^−*/*−^ mice is of particular interest. If observed in isolation, this phenomenon might lead to overactivation of pyramidal neurons and damage, but the same effect could prove protective in the context of LOAD-associated factors that *reduce* neuronal function. The protective effect of TYROBP deficiency in an early AD context is confirmed by the improvement in the behavioral performance of *APP/PSEN1* mice deficient in TYROBP. The effect of the *Tyrobp*-null background on the electrophysiological findings and gene set enrichment (synapse assembly, ion transport, and neurotransmission) of *APP/PSEN1; Tyrobp* ^−*/*−^ vs. *APP/PSEN1* are in keeping with the growing appreciation for the role of microglia in maintaining normal synaptic physiology [4]. Indeed, in addition to their pro-inflammatory and phagocytic functions, microglia release cytokines including TGFβ and interleukin-1β that acutely modulate synaptic plasticity at hippocampal synapses [11, 71].

Thus, in a comprehensive panel of transcriptomic, biochemical, electrophysiological, and behavioral paradigms, reduction or ablation of TYROBP prevented the expression of many of the corresponding *APP/PSEN1* phenotypes at 4 months of age. These results would appear to argue against the possibility that early TYROBP deficiency is likely to be a predisposing factor for LOAD. Indeed, these results would indicate that a decrease in TYROBP activity could represent an important therapeutic opportunity either for treating or preventing LOAD or else for slowing or arresting the progression of MCI or early AD to full-blown clinical and pathological LOAD.

## Electronic supplementary material

Below is the link to the electronic supplementary material.

Suppl. Figure 1: Deficiency or absence of TYROBP does not modify microglia number in 4-month-old WT mice. (a-b) Images of NISSL (a) and Iba1 staining (b) in prefrontal cortices (PFC) and hippocampi of 4-month-old *Tyrobp*(+/+), *Tyrobp*(+/-) and *Tyrobp*(-/-) mice. (c-d) Flow cytometry analysis of CD45^mid^ and CD11b^high^ cells (microglia) of 4-month-old *Tyrobp*(+/+) (n=3) and *Tyrobp*(-/-) (n=7) mouse brains. Representative flow cytometry plots after live/dead and doublet cells exclusions showing CD45^mid^ and CD11b^high^ cells (blue squares) in *Tyrobp*(+/+) and *Tyrobp*(-/-) mouse brains (c). Percentages of the microglia population are presented in (d). (TIFF 7851 kb)

Suppl. Figure 2: Female *APP/PSEN1;Tyrobp* ^*-/-*^ mice have lower Aß40 levels than their counterparts with normal levels of *Tyrobp.* (a-i) Hemibrains of male and female *APP/PSEN1* (n=4-6), *APP/PSEN1;Tyrobp* ^*+/-*^ (n = 3-8) and *APP/PSEN1;Tyrobp* ^*-/-*^ (n=3-4) mice were processed via differential detergent solubilization to produce fractions of TBS soluble, Triton-X soluble, and formic acid soluble Aβ. Levels of Aß40 (a-c) and Aß42 (d-f) were determined from each fraction via ELISA. The Aβ42/40 ratio was calculated for each fraction (g-i). (TIFF 756 kb)

Suppl.3_DEG_Tyrobp.vs.WT (XLSX 51 kb)

Suppl.4_DEG_APPPSEN1;Tyrobp (XLSX 97 kb)

Suppl.5_GSEA_APPPSEN1;Tyrobp mice (XLSX 56 kb)

Suppl.6_APPPSEN1;Tyrobp.mice.network (XLSX 12 kb)
