# Supplementary figures and images for: Deficiency of TYROBP, an adapter protein for TREM2 and CR3 receptors, is neuroprotective in a mouse model of early Alzheimer’s pathology
Source: Acta Neuropathol. 2017 Jun 13;134(5):769–88. doi: 10.1007/s00401-017-1737-3 (PMC5645450; doi:10.1007/s00401-017-1737-3)

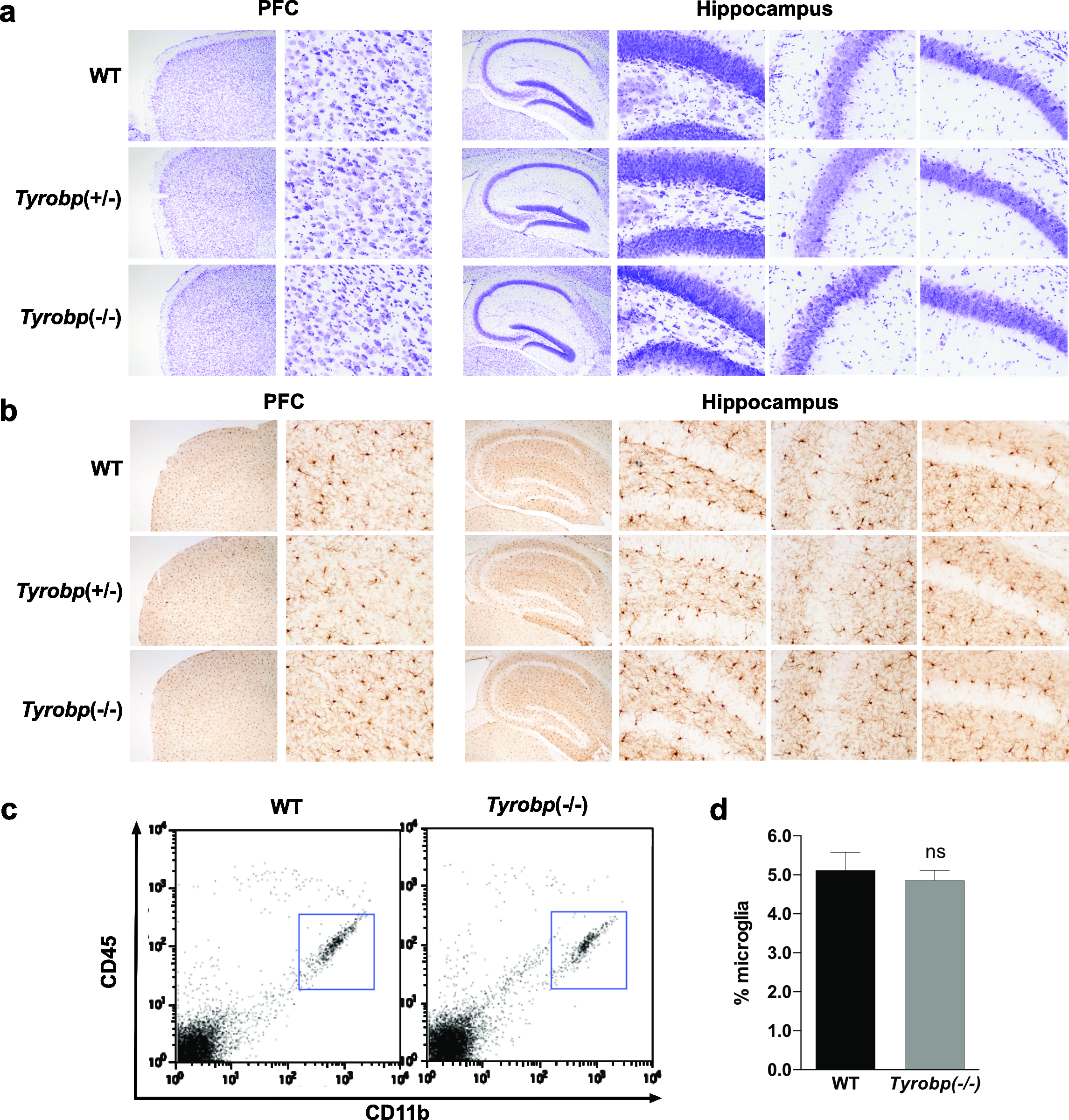

Supplement: Supplementary file 1 — Suppl. Figure 1: Deficiency or absence of TYROBP does not modify microglia number in 4-month-old WT mice. (a-b) Images of NISSL (a) and Iba1 staining (b) in prefrontal cortices (PFC) and hippocampi of 4-month-old Tyrobp(+/+), Tyrobp(+/-) and Tyrobp(-/-) mice. (c-d) Flow cytometry analysis of CD45mid and CD11bhigh cells (microglia) of 4-month-old Tyrobp(+/+) (n=3) and Tyrobp(-/-) (n=7) mouse brains. Representative flow cytometry plots after live/dead and doublet cells exclusions showing CD45mid and CD11bhigh cells (blue squares) in Tyrobp(+/+) and Tyrobp(-/-) mouse brains (c). Percentages of the microglia population are presented in (d). (TIFF 7851 kb) [file 401_2017_1737_MOESM1_ESM.tiff]

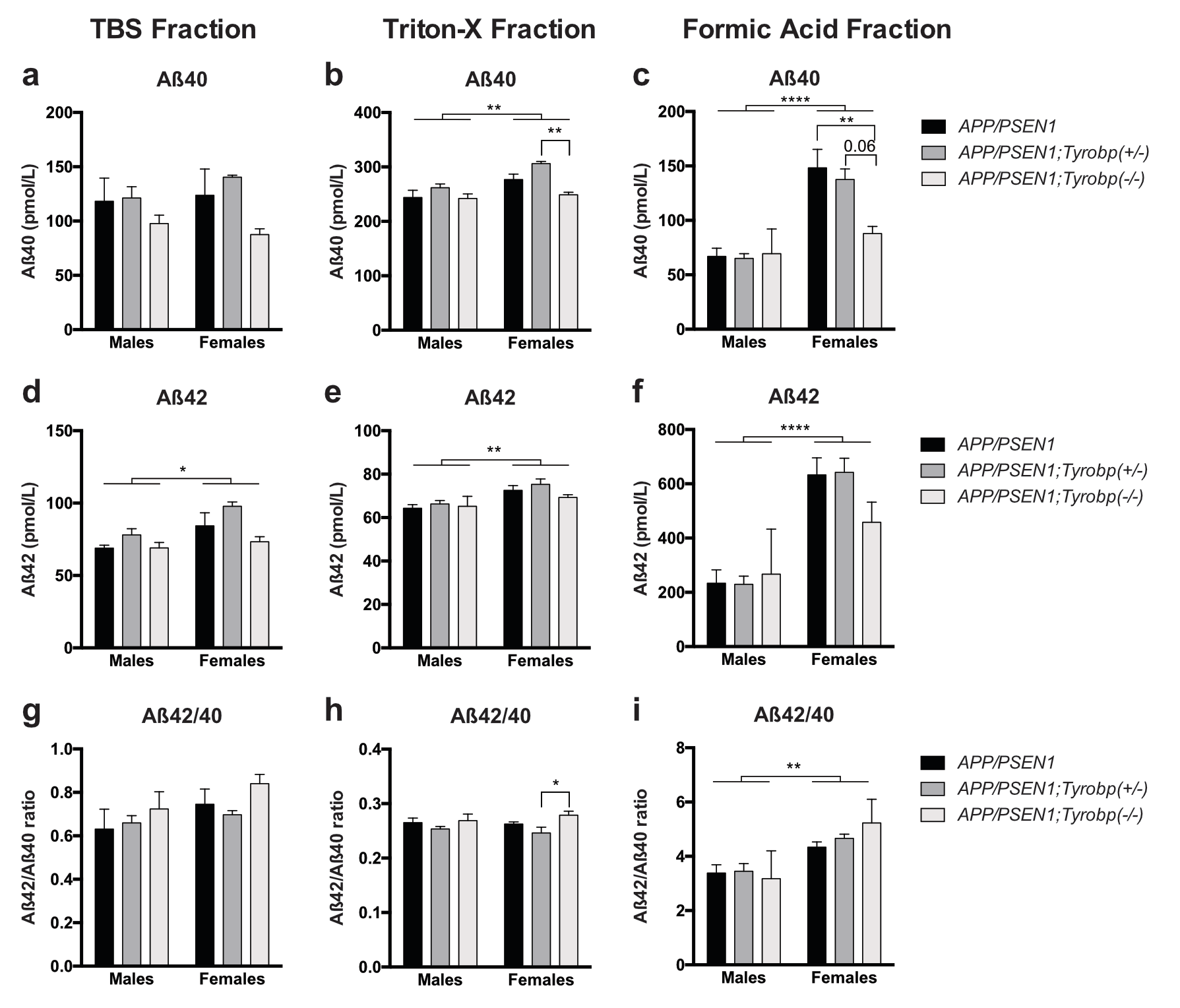

Supplement: Supplementary file 2 — Suppl. Figure 2: Female APP/PSEN1;Tyrobp -/- mice have lower Aß40 levels than their counterparts with normal levels of Tyrobp. (a-i) Hemibrains of male and female APP/PSEN1 (n=4-6), APP/PSEN1;Tyrobp +/- (n = 3-8) and APP/PSEN1;Tyrobp -/- (n=3-4) mice were processed via differential detergent solubilization to produce fractions of TBS soluble, Triton-X soluble, and formic acid soluble Aβ. Levels of Aß40 (a-c) and Aß42 (d-f) were determined from each fraction via ELISA. The Aβ42/40 ratio was calculated for each fraction (g-i). (TIFF 756 kb) [file 401_2017_1737_MOESM2_ESM.tiff]
